# RETRACTED ARTICLE: Relationship between smoking and serum levels of eye muscle and orbital connective tissue antibodies in patients with Graves ophthalmopathy

**DOI:** 10.1007/s12020-023-03335-5

**Published:** 2023-03-11

**Authors:** Hooshang Lahooti, Bernard Champion, Jack R. Wall

**Affiliations:** 1 Department of Medicine, The University of Sydney, Nepean Clinical School, Sydney, NSW Australia; 2 Department of Health Sciences, Macquarie University, Sydney, NSW Australia

The Editor-in-Chief has retracted this paper because the authors did not seek informed consent from participants prior to conducting the telephone interviews. Concerns have also been raised about the accuracy of some of the demographic information reported in this study. The authors do not have access to the original data, and were unable to confirm the accuracy of the information reported. The Editor-in-Chief therefore no longer has confidence in the reliability of the data. Jack R Wall agrees with this retraction. The remaining authors did not respond to correspondence from the publisher about this retraction. The online version of this article contains the full text of the retracted article as Supplementary Information.

### Supplementary information

Former article version

