# Supplementary material for: RETRACTED ARTICLE: Relationship between smoking and serum levels of eye muscle and orbital connective tissue antibodies in patients with Graves ophthalmopathy
Source: Endocrine. 2023 Mar 11;85(1):459. doi: 10.1007/s12020-023-03335-5 (PMC11246247; doi:10.1007/s12020-023-03335-5)
Supplement: Supplementary file 1 — Former article version [file 12020_2023_3335_MOESM1_ESM.pdf]

Endocrine
https://doi.org/10.1007/s12020-023-03335-5

ORIGINAL ARTICLE

Relationship between smoking and serum levels of eye muscle and orbital connective tissue antibodies in patients with Graves ophthalmopathy

Hooshang Lahooti[1] · **Bernard Champion**[2] · **Jack R. Wall**[1,2]

Received: 22 March 2022 / Accepted: 19 February 2023
© Crown 2023

Abstract

Over the past three decades, several studies have quantified the risk of smoking in the development of ophthalmopathy in patients with Graves' hyperthyroidism, with an overall odds ratio of approximately 3.0. Smokers also have a greater risk of more advanced ophthalmopathy than non-smokers. We studied 30 patients with Graves' ophthalmopathy (GO) and 10 patients with upper eyelid signs as the only manifestation of ophthalmopathy, whose eye signs were assessed using the clinical activity score (CAS), NOSPECS classes and upper eyelid retraction (UER) score, half of whom were smokers and half of whom were non-smokers. Serum levels of eye muscle (CSQ, Fp2, G2s) and orbital connective tissue type XIII collagen (Coll XIII) antibodies are valuable markers of ophthalmopathy in patients with Graves' disease. Still, their relationship to smoking has not been investigated. These antibodies were measured by enzyme-linked immunosorbent assay (ELISA) in all patients as a component of their clinical management. Mean serum antibody levels of all four antibodies were significantly greater in smokers than in non-smokers in patients with ophthalmopathy but not in those with upper eyelid signs only. As determined using one-way ANOVA and Spearman's correlation test, there was a significant correlation between smoking severity, assessed as pack-years, with mean Coll XIII antibody level, but not with levels of the 3 eye muscle antibodies. These results suggest that in patients with Graves' hyperthyroidism who smoke, the orbital inflammatory reactions are more advanced than in those with Graves' hyperthyroidism who do not smoke. The mechanism of this enhanced Autoimmunity against orbital antigens in smokers is unclear and worthy of further study.

Keywords Ophthalmopathy · Graves' hyperthyroidism · Eye muscle antibodies · Calsequestrin · Type XIII collagen · Upper eyelid retraction

RETRACTED ARTICLE

Introduction

Eye changes are found in about 50% of patients with Graves' hyperthyroidism [1] and less often in patients with Hashimoto's thyroiditis [1, 2]. The various eye and orbital signs and symptoms are characterized as Graves' ophthalmopathy, also called Graves' orbitopathy (GO). Various antibodies are linked with the ophthalmopathy, of which those targeting the thyroid-stimulating hormone receptor (TSHR) [3, 4] and the insulin-like growth factor-1 receptor (IGF1-R) [5, 6] may be the best candidates to play an etiological role in this complex and controversial eye and orbital disorder.

Antibodies targeting the calcium-binding protein calsequestrin (CSQ), which is expressed five times more in eye muscle than other skeletal muscle [7], are found in the majority of patients with recent-onset Graves' hyperthyroidism and orbital inflammatory changes [8–10] and therefore should also be considered candidates. In earlier studies, we also identified serum antibodies against; the so-called "64 kDa protein", shown to be the flavoprotein (Fp2) subunit of the mitochondrial succinate dehydrogenase [11–13], G2s, the terminal region of the transcription factor of FOXP1 [14, 15] and the connective tissue protein type XIII collagen (Coll XIII) [16, 17].

✉ Hooshang Lahooti
hooshang.lahooti@sydney.edu.au

1 Department of Medicine, The University of Sydney, Nepean Clinical School, Sydney, NSW, Australia

2 Department of Health Sciences, Macquarie University, Sydney, NSW, Australia

RETRACTED ARTICLE

While the three eye muscle antibodies (CSQ, Fp2 and G2s) are all markers of ophthalmopathy in patients with Graves' disease, they are probably secondary to the eye muscle reactions as they are all intracellular. On the other hand, Coll XIII is an extracellular transmembrane protein in the orbital fibroblast and, therefore, a candidate for a target antigen for the orbital connective tissue (OCT) component of ophthalmopathy.

Cigarette smoking affects thyroid morphology and function [18, 19]. Several lines of research have demonstrated the role of cigarette smoking in the development of ophthalmopathy in patients with Graves' hyperthyroidism. Ophthalmopathy is found in about 60% of smokers but only 20% of non-smokers [20, 21]. Moreover, smokers with ophthalmopathy tend to have more severe diseases than non-smokers with eye disorders [22]. Smokers with GO have a lower and slower response to immunosuppressive treatment than non-smokers [23]. GO patients who stopped smoking have a lower risk of developing exophthalmos and diplopia than current smokers [24]. In recent years the prevalence of GO has been declining in European countries where tobacco consumption has decreased [25].

In our studies over many years, we have studied the pathogenesis of GO in experimental and clinical studies. Here, we have addressed a possible relationship between smoking and serum levels of antibodies against CSQ, G2s, Fp2 and type XIII collagen in patients with Graves' ophthalmopathy who smoked compared to those who did not smoke. Our findings provide evidence for the role of orbital Autoimmunity against these antigens in the pathogenesis of the orbital reactions that can be further studied in the future in animal models for GO.

Experimental methods

Clinical subjects

We studied 30 patients with Graves' ophthalmopathy, 8 males and 22 females aged 20–79 (mean age 46 yr.), of whom 4 males and 11 females were smokers and 4 males and 11 females were non-smokers and 10 patients, all women aged 26 to 54 (mean age 44 yr.) with upper eyelid signs as the only manifestation of ophthalmopathy, of whom 5 smoked and 5 did not smoke Ophthalmopathy was defined as NOSPECS classes 2 or more [26], i.e., all patients had at least chemosis, conjunctival injection and periorbital swelling (NOSPECS classes 1–3), and some had eye muscle involvement (double vision, reduced upward gaze (NOSPECS class 4). Patients with only upper eyelid retraction or lag (NOSPECS class 1, only signs) were included as a separate group, termed "upper eyelid changes only".

All patients with ophthalmopathy had moderately severe or severe eye disease, which was active, quantified as the clinical activity score (CAS) of Moritz et al. [27] of 3 or more. Patients were randomly selected from representative patients seen at the Nepean Hospital Thyroid clinics and JW private practice. During the period 2005–2015.

Inclusion criteria were; patients with proven GO whose smoking status was known, aged 18–80. Exclusion criteria were; age <18 (whose smoking history might be unreliable) or >80, treatment with steroids or immunosuppressive drugs, or refusal to provide their smoking history and status.

The patient serum antibody results were obtained from their clinical files. Eye muscle and Coll XIII antibody tests were carried out in the context of their clinical management. The patient's smoking status (yes, no, no. of pack years) was obtained by telephone. The Nepean Blue Mountains Human Ethics Committee approved the study (Code: study 08/017-08/NEPEAN/20, Approval Date: 10 July 2014) and the telephone contact with patients to establish their smoking status. As far as we know, none of the subjects in the study was passive smokers. The patient's blood samples were studied in the routine management of patients with Graves disease with and without ophthalmopathy, and antibody assays were performed as necessary. Because the study was retrospective and anonymous, consent forms were not needed.

Enzyme-linked immunosorbent assay (ELISA)

The method has been described in previous publications from this laboratory [8–10]. Tests were performed in triplicate in 96 wells plates. In preliminary assays, the optimal concentrations of purified CSQ, Fp2, G2s and Coll XIII were found to be one pg/ml for each protein, and optimal serum dilutions were 1/50 for CSQ, Fp2, G2s, and 1/25 for Coll XIII, respectively. The second antibody was an alkaline phosphatase· labelled goat anti-human IgG diluted 1/4000. We used phosphate-buffered saline (PBS) as a control instead of antigen, serum or secondary antibody. Results were expressed as optical density (OD) at 410 NM using an Optima plate reader and a positive test taken as an OD > mean +2 SD for a panel of age and sex-matched healthy subjects.

Statistical analysis

Mean serum levels of the four orbital antibodies were compared for smokers and non-smokers using repeated measures of one-way analysis of variance (ANOVA) with Geisser- Greenhouse correction. The correlation between antibody levels and smoking status and severity was assessed using Spearman's correlation coefficient. For all analyses, a P-value of < 0.05 was considered significant.

Table 1 Mean eye muscle and orbital connective tissue antibody levels in patients with Graves' ophthalmopathy or isolated upper eyelid signs who smoked compared to patients with Graves' ophthalmopathy or isolated upper eyelid signs who did not smoke

Group *N*	Mean antibody levels in patients with ophthalmopathy[a]				Group *N*	Mean antibody levels in patients with upper eyelid signs only			
	CSQ	Fp2	G2s	Coll XIII		CSQ	Fp2	G2s	Coll XIII
Smoker ($n = 15$)	157.2	188.4	152.2	228.7	Smoker ($n = 5$)	154.8	198.8	116.5	301.5
Non-smoker ($n = 15$)	139.3	146.7	91.9	189.2	Non-smoker ($n = 5$)	144.6	218.5	113.9	298.2
[a]*P*-values	0.0200				[a]*P*-values	0.888			
[b]*P*-values	0.152				[b]*P*-values	0.0513			

CSQ calsequestrin, *Fp2* flavoprotein subunit of succinate dehydrogenase, *G2s* the terminal fragment of the FOXP1 transcription factor, *Coll XIII* collagen type XIII

[a]Statistical analyses compare smokers and non-smokers (rows above table) for each antibody, as described in experimental methods using repeated measures one-way ANOVA with Geisser- Greenhouse correction

[b]Statistical analyses comparing smokers and non-smokers between the groups (columns in the above table) in reference to each studied antibody using repeated measures one-way ANOVA with Geisser- Greenhouse correction

Table 2 Correlation between smoking severity was assessed as pack years and respective serum antibody levels in patients with Graves' ophthalmopathy or isolated upper eyelid signs who smoked

Statistical test	Correlations of pack years and respective serum antibody levels in smokers with Graves' ophthalmopathy ($n = 15$) or isolated upper eyelid signs ($n = 5$) taken as a single group			
	CSQ	Fp2	G2s	Coll XIII
Spearman's correlation coefficient	0.209	−0.0604	−0.143	−0.599
P value	0.481	0.835	0.629	0.02889

CSQ calsequestrin, *Fp2* flavoprotein subunit of succinate dehydrogenase, *G2s* the terminal fragment of the FOXP1 transcription factor, *Coll XIII* collagen type XIII. Statistical analyses were carried out using Spearman's test

RETRACTED ARTICLE

The GraphPad Prism 8 statistical package was used for the analyses.

Results

We studied patients with Graves ophthalmopathy ($n = 30$) or isolated upper eyelid lag and retraction ($n = 10$), of whom half were smokers, and half were non-smokers. The number of cigarettes smoked was quantified as cigarette pack years. The mean serum antibody levels were compared between smokers and non-smokers for each CSQ, Fp, G2s and Coll XIII. The statistical significances between groups for each of the 4 antibodies were assessed using repeated measures of one-way ANOVA with Geisser- Greenhouse correction. The results are summarized in Tables 1 and 2.

As seen in Table 1, mean serum antibody levels of all four antibodies were greater in smokers than non-smokers in patients with ophthalmopathy and, narrowly, but not significantly, in 3 of the 4 antibodies in patients with eyelid signs only. When statistical significance between groups in reference to each studied antibody was compared for smokers and non-smokers, there was no significant effect of smoking on the mean of each antibody ($P = 0.152$ for the ophthalmopathy group and $P = 0.0513$, which failed to reach the significance of the upper eyelids sign. When comparing the mean antibody for smokers and non-smokers (comparing the rows of smokers to non-smokers), there was a significant difference between the means for ophthalmopathy patients ($P = 0.0200$); however, there was no significant difference for patients with isolated upper eyelids sign patients ($P = 0.888$).

To show the association of smoking on the mean level of eye muscles antibodies in ophthalmology and upper eyelids retraction groups using the Brown-Forsythe and Welch ANOVA test showed a significant difference between the mean level of eye muscles antibodies ([$P = 0.0211$] data not shown). We also used the one-sample *t*-test to analyze the relationship between mean antibody levels and smoking for each antibody, namely; for the CSQ antibody; $P = 0.0387$, which is significant; for the Fp2 antibody, $P = 0.0796$, which is not significant; for G2s antibody $P = 0.1566$ which is not significant and for Coll XIII antibody $P = 0.0594$ which just failed to reach significance (results not shown).

Finally, we correlated smoking severity, measured as pack-years, with mean serum antibody levels in smokers

with ophthalmopathy or upper eyelid signs only (Table 2). There were no significant correlations between the amount of cigarette smoking and serum orbital antibody level for CSQ, Fp2 and G2s antibodies; however, there was a significant correlation for Coll XIII antibodies $P = 0.02889$ (Spearman's correlation coefficient, $P > 0.05$ for the other 3 antibodies.

Discussion

The pathogenesis of GO is not entirely understood. Still, it most likely involves an antibody reaction against the IGF-1R that activates T cells against antigens in the retro-orbital space that share antigenic epitopes with proteins in the thyroid follicular cells. These immune processes lead to orbital inflammation, with lymphocyte infiltration of the orbital tissues, including the eye muscles and the release of cytokines that stimulate orbital fibroblasts to multiply and produce glycosaminoglycans, which absorb water, leading to an increase in volume.

Smoking is a significant risk factor for ophthalmopathy in patients with Graves' disease. Overall, the prevalence of ophthalmopathy in patients with newly diagnosed Graves' hyperthyroidism who smoke is approximately 60% compared to 20% in non-smokers [18–21]. In addition, patients who smoke appear to be more likely to experience worsening of their ophthalmopathy if treated with radioactive iodine [22]. In the present study, we have correlated status and severity with positivity and levels of serum autoantibodies against 4 eye muscle and orbital connective tissue antigens known to be markers, if not the cause, of the ophthalmopathy.

To summarize the main results, serum antibody levels for all four antibodies were greater in smokers than non-smokers in patients with ophthalmopathy and for 3 of the 4 antibodies in patients with eyelid signs only and G2S proteins, which are intracellular proteins, but the effect is not significant.

Detailed statistical analysis in this report showed a strong association of smoking with Graves' ophthalmopathy but not in patients with isolated upper eyelids retraction.

The possible mechanisms whereby smoking affects GO are complex and involve several factors. Smoke-induced increased generation of oxygen free radicals and hypoxia can stimulate orbital fibroblasts to proliferate and synthesize glycosaminoglycans [23]. In the presence of interferon-γ, nicotine enhances HLA-DR expression in cultured orbital fibroblasts [24]. Cultured orbital cells exposed to smoke extracts; adipogenesis increased, and this effect was synergistic with IL-1 [25]. In untreated GO, they smoke increased extraocular muscle volume rather than fat [28, 29]. It seems likely, however, that the association is due to a complex, genetically based but poorly understood neuro-immunological process that is enhanced in smokers.

As can be seen in Table 2, the number of cigarettes smoked yearly increases, the Coll XIII antibody levels decrease. This is significant because the level of Coll XIII protein which is a cell surface membrane-bound protein, may be increased in the tissue fluid surrounding the B-cells which results in an initial increase in antibody production, but chronic or continuous exposure to cigarette smoke causes downregulation of surface Coll XIII by cellular processes and less Coll XIII protein becomes available for stimulation of Coll XIII antibody production. As seen in Table 2, there is a negative correlation between antigens and corresponding antibodies for Fp2 and G2s.

The results of our study suggest that our approach is an appropriate way to address the effect of smoking on orbital autoimmune reactions. Of interest is that this association was shown for patients with ophthalmopathy. This was not the case for patients with upper eyelid signs. This suggests that eyelid lag and retraction, although major features of Graves' ophthalmopathy [1], are not caused by orbital inflammation and may have different pathogenesis.

Recent studies [5, 6] have demonstrated that autoimmunity against the IGF-1R alone or in conjunction with the TSHR may be the best candidate for a specific etiological reaction that can explain the development of ophthalmopathy in patients with Graves' disease. A similar approach could be used to examine the role of smoking in targeting these two proteins in orbit.

Limitations of our study

The main limitations of our study are (i) the small numbers of patients were studied; the study was partly retrospective and partly prospective; (ii) although pack-years can be taken as a reliable estimate of tobacco consumption during the time such self-reporting information could be subject to recall bias [27, 30] (iii) because of lack of operative or biopsy orbital tissue samples we were not able to correlate the antibody results with histological evidence for orbital tissue infiltration and damage.

Conclusions

Our findings provide evidence for the role of Autoimmunity against these antigens in the pathogenesis of the orbital reactions that can be further studied in the future in animal models for GO. The results suggest that in patients with Graves' hyperthyroidism who smoke, the orbital inflammatory reactions, measured as mean serum levels of antibody against candidate orbital autoantigens, are more significant than in patients with Graves' hyperthyroidism who do not smoke. The mechanism of this enhanced

RETRACTED ARTICLE

RETRACTED ARTICLE

Autoimmunity against orbital antigens in smokers is unknown but worthy of further study.

Author contributions J.W. and B.C. developed the hypothesis, collected the patients' information and wrote the original manuscript draft; H.L. performed the laboratory tests and statistical analysis and helped write the revision; J.W. wrote the final version of the revision, which all authors have read. All authors have read the final version of the manuscript and agree that it is ready for the Journal.

Funding Open Access funding enabled and organized by CAUL and its Member Institutions.

Compliance with ethical standards

Conflict of interest The authors declare no competing interests.

Ethics The Nepean Mountains Human Ethics Committee approved the study and the telephone contact with patients to establish their smoking status. Because the study was retrospective and anonymous, consent forms were not needed. The study was conducted following the Declaration of Helsinki and approved by the Institutional Review Board of The University of Sydney.

Publisher's note Springer Nature remains neutral with regard to jurisdictional claims in published maps and institutional affiliations.

Open Access This article is licensed under a Creative Commons Attribution 4.0 International License, which permits use, sharing, adaptation, distribution and reproduction in any medium or format, as long as you give appropriate credit to the original author(s) and the source, provide a link to the Creative Commons license, and indicate if changes were made. The images or other third party material in this article are included in the article's Creative Commons license, unless indicated otherwise in a credit line to the material. If material is not included in the article's Creative Commons license and your intended use is not permitted by statutory regulation or exceeds the permitted use, you will need to obtain permission directly from the copyright holder. To view a copy of this license, visit http://creativecommons.org/licenses/by/4.0/.

References

1. R.S. Bahn, Current Insights into the Pathogenesis of Graves' Ophthalmopathy. Horm. Metab. Res **47**, 773–773 (2015)
2. H. Tjiang, H. Lahooti, T. McCorquodale, K.R. Parmar, J.R. Wall, Eye and eyelid abnormalities are common in patients with Hashimoto's thyroiditis. Thyroid **20**, 287–290 (2010)
3. H.B. Burch, L. Wartofsky, Graves' ophthalmopathy: current concepts regarding pathogenesis and management. Endocr. Rev. **14**, 747–793 (1993)
4. W. Wiersinga, Autoimmunity in Graves' ophthalmopathy: the result of an unfortunate marriage between TSH receptors and IGF-1 receptors? J. Clin. Endocrinol. Metab. **96**, 2386–2394 (2011)
5. C.C. Krieger, R.F. Place, C. Bevilacqua, B. Marcus-Samuels, B.S. Abel et al. TSH/IGF-1 receptor cross-talk in Graves' ophthalmopathy pathogenesis. J. Clin. Endocrinol. Metab. **101**, 2340–2347 (2016)
6. T.J. Smith, J. Jansen, Insulin-like growth factor-I receptor and thyroid-associated ophthalmopathy. Endocr. Rev. **40**, 236–267 (2019)
7. L. Wescombe, B. Gopinath, H. Lahooti, J.R. Wall, The cardiac calsequestrin gene (CASQ2) is up-regulated in the thyroid in patients with Graves' ophthalmopathy - support for a role of Autoimmunity against calsequestrin as the triggering event. Clin. Endocrinol. (Oxf.) **73**, 522–528 (2010)
8. K. Gunji, S. Kubota, C. Stolarski, S. Wengrowicz, J.S. Kennerdell et al. A 63 kDa skeletal muscle protein associated with eye muscle inflammation in Graves' disease is identified as the calcium-binding protein calsequestrin. Autoimmunity **29**, 1–9 (1999)
9. B. Gopinath, J. Tani, N. Bao, J.R. Wall, Eye signs and serum eye muscle and collagen XIII antibodies in patients with transient and progressive thyroiditis. Thyroid **17**, 1–7 (2007)
10. B. Gopinath, Wescombe, B. Nguyen, J.R. Wall, Can Autoimmunity against calsequestrin explain the eye and eyelid muscle inflammation of thyroid eye disease? Orbit **28**, 256–261 (2009)
11. M. Salvi, A. Miller, J.R. Wall, Human orbital tissue and thyroid membranes express a 64-kDa protein, which is recognized by autoantibodies in the serum of patients with thyroid-associated ophthalmopathy. FEBS Lett. **232**, 135–139 (1988)
12. M. Salvi, H. Fukazawa, Y. Hiromatsu, H. Triller, N. Bernard et al. Role of autoantibodies in the pathogenesis of endocrine autoimmune disorders and in their association. Endocr. Rev. **9**, 450–466 (1988)
13. K. Gunji, A. De Bellis, S. Kubota, J. Swanson, S. Wengrowicz et al. Serum antibodies reactive against the flavoprotein subunit of protein subunit of succinate dehydrogenase are sensitive markers of eye muscle autoimmunity in patients with Graves' hyperthyroidism. J. Clin. Endocrinol. Metab. **84**, 16–22. (1999)
14. K. Gunji, A.M. De Bellis, A.W. Li, M. Yamada, S. Kubota et al. Cloning and characterization of G2s, a novel eye muscle and thyroid shared autoantigen associated with the development of ophthalmopathy in patients with thyroid autoimmunity. J. Clin. Endocrinol. Metab. **85**, 1641–1647 (2000)
15. H.-S. Chang, J. Tani, A.W. Li, J.R. Wall, A patient with Graves' disease in whom presenting eye signs were associated with antibodies to the Thyrotropin receptor and later eye muscle damage with antibodies to G2s and flavoprotein. Int. J. Endo Metab. **1**, 143–148 (2005)
16. B. Gopinath, C.J. Adams, R. Musselman, J. Tani, J.R. Wall, Antibodies against calsequestrin and type XIII collagen are good markers for chronic upper eyelid lag and retraction. Ocul. Immunol. Inflamm. **15**, 81–88 (2007)
17. B. Gopinath, R. Musselman, C. Adams, J. Tani, N. Beard, J.R. Wall, Study of serum antibodies against three eye muscle antigens and the connective tissue antigen collagen XIII in patients with Graves' disease with and without ophthalmopathy – correlation with clinical features. Thyroid **16**, 967–974 (2006)
18. L. Bartalena, F. Bogazzi, M.L. Tanda, L. Manetti, E. Dell' Unto, Cigarette smoking and the thyroid. Eur. J. Endocrinol. **133**, 507–512 (1995)
19. N. Pontikides, G.E. Krassas, Influence of cigarette smoking on thyroid function, goiter formation and autoimmune thyroid disorders. Hormones **1**, 91–98 (2002)
20. J. Thornton, S.P. Kelly, R.A. Harrison, R. Edwards, E. Hägg et al. Is endocrine ophthalmopathy related to smoking? Br. Med J. **295**, 634–635 (1987)
21. L. Hegedüs, T.H. Brix, P. Vestergaard, Relationship between cigarette smoking and Graves' ophthalmopathy. J. Endocrinol. Invest **27**, 265–271 (2004)
22. J. Thornton, S.P. Kelly, R.A. Harrison, Edwards, Cigarette smoking and thyroid eye disease: a systematic review. Cigar. Smok. Thyroid Eye Dis.: Syst. Rev. JAMA Ophthalmol. **132**, 253–257 (2014)
23. R.M. Rui, P.W. Lu, L. Wartofsky, B.D. Sutton, J.I. Zwieir et al. Oxygen free radicals in interleukin-1β-induced glycosaminoglycan

production by retro-ocular fibroblasts from normal subjects and Graves' ophthalmopathy patients. Thyroid **9**, 297–303 (2009)

24. W.P. Mack, G.O. Stasior, H.J. Cao, O.G. Stasior, T.J. Smith, The effect of cigarette smoke constituents on the expression of HLA-DR in orbital fibroblasts derived from patients with Graves ophthalmopathy. Ophthalmic Plast. Reconstr Surg. **15**, 260–271 (1999)
25. G.O. Stasior, H.J. Cao, T.J. Smith, The effect of cigarette smoking constituents on the expression of HLA-DR in orbital fibroblasts derived from patients with Graves ophthalmopathy. Ophthalmic Plast. Reconstr Surg. **15**, 260–271 (1999)
26. S.C. Werner, Classification of the eye changes of Graves' disease. J. Clin. Endocrinol. Metab. **1969**(29), 982–984 (1969)
27. M.P. Mourits, M.F. Prummel, W.M. Wiersinga, Koornneef, Clinical activity score as a guide in the management of patients with Graves' ophthalmopathy. Clin. Endocrinol. **47**, 9–14 (1997)
28. N.I. Regensburg et al. Effect of smoking on orbital fat and muscle volume in Grave' orbitopathy (2010). Published Online: https://doi.org/10.1089/thy.0218
29. R.A. Metcalfe, A.P. Weetman, Stimulation of extraocular muscle fibroblasts by cytokines and hypoxia: possible role in thyroid-associated ophthalmopathy. Clin. Endocrinol. **40**, 67–72 (1994).
30. E. Hirvonen, M. Stepanov, M. Kilpeläinen, A. Lindqvist, T. Laitinen, Consistency and reliability of smoking-related variables: longitudinal study design in asthma and COPD. Eur. Clin. Respir. J. **6**, 1 (2019).

RETRACTED ARTICLE
